# Drug supply shortages and their perceived consequences for patients: a questionnaire survey of German and Austrian physicians

**DOI:** 10.1007/s00228-026-04052-4

**Published:** 2026-05-01

**Authors:** Julia Maria Rotter, Roland Seifert

**Affiliations:** Institute of Pharmacology, Hannover Medical School, Carl-Neuberg-Straße 1, Hannover, D-30625 Germany

**Keywords:** Supply shortage, Supply bottleneck, Medicine shortage, Antimicrobial resistance

## Abstract

**Purpose:**

Drug supply shortages are a recurring issue in developed countries, with consequences for patients even before the COVID-19 pandemic. Questions arise regarding the effectiveness and tolerability of alternative treatments chosen by physicians due to these shortages.

**Methods:**

To answer these questions, a survey for practicing physicians was distributed to medical associations in Germany and Austria and conducted from November 2022 to January 2024. 895 physicians responded to the survey. The survey targeted 20 drugs with known supply shortages, namely amoxicillin, amoxicillin/clavulanic acid, penicillin V (phenoxymethylpenicillin), cefuroxime, cefaclor, erythromycin, cotrimoxazole, ibuprofen, paracetamol, urapidil, metoprolol, amlodipine, candesartan, tamoxifen, methotrexate, fluoxetine, lorazepam, human insulin, salbutamol and prednisolone.

**Results:**

Physicians most frequently chose a different antibacterial drug (> 60% of the physicians), while for analgesics, they more often used a different dosage form of the same drug (> 33%). For antihypertensive drugs, physicians more often chose a different dosage of the same drug. In many cases, alternative antibiotics were chosen that carried a greater risk of antimicrobial resistance than the antibiotic originally intended. The treatment success for replacing antibacterials and analgesics with a different drug was rated with 4–5 on a predefined scale of 1 (very poor) to 6 (very good) in comparison to the original drug. Using the same drug in a different dosage/dosage form was also around 4–5/6 effective.

**Conclusions:**

Supply shortages can foster antimicrobial resistance through the use of antibacterials with a higher potential for resistance. The success of alternative treatments was not always considered to be very good in comparison to the original medication.

**Supplementary Information:**

The online version contains supplementary material available at 10.1007/s00228-026-04052-4.

## Introduction

In Germany and Austria, patients are affected by drug shortages. In a member survey conducted by the Association of Statutory Health Insurance Physicians (“Kassenärztliche Vereinigung”) Berlin in August 2023, 81% of physicians said their patients had problems receiving the medication they prescribed from a pharmacy. In addition, 54% of physicians reported that patients in their practices are increasingly asking for prescriptions in order to be well supplied with medication for the next few months [1]. A representative population survey carried out on behalf of PHAGRO (Bundesverband des pharmazeutischen Großhandels e.V., Federal Association of Pharmaceutical Wholesalers [in Germany]) in September and October 2024 showed that one in two are aware of drug supply shortages from their daily life. 29% of survey participants have personally experienced a situation where a required medication was not available at the pharmacy and could not be ordered. 22% stated this had occurred in their immediate family [2]. In Austria, around one in six people are affected by supply shortages [3].

The objectives of the present work were to obtain information on how these drug supply shortages are handled by physicians and what effects on patient’s treatment they perceived.

The following evaluation of a questionnaire survey, completed by physicians practicing in Germany and Austria, addresses respective consequences resulting from supply shortages of 20 selected drugs.

## Materials and methods

### General information about the creation of the survey

The survey focuses on the supply shortages and their consequences for patients of 20 selected drugs, including amoxicillin, amoxicillin/clavulanic acid, penicillin V (phenoxymethylpenicillin), cefuroxime, cefaclor, erythromycin, cotrimoxazole, ibuprofen, paracetamol, urapidil, metoprolol, amlodipine, candesartan, tamoxifen, methotrexate, fluoxetine, lorazepam, human insulin, salbutamol, and prednisolone. The count of 20 drugs was chosen arbitrarily, with the intention to ensure that the dataset is sufficient to gain a good understanding of the consequences of the supply shortage while keeping the workload of the survey participant to a minimum.

All the drugs listed, except urapidil and cefaclor, can be found in the WHO (World Health Organization) Model List of Essential Medicines [4]. The European Union list of critical medicines published by EMA (European Medicines Agency) also includes most of the 20 drugs. Not included are urapidil, cefaclor, ibuprofen, metoprolol, amlodipine, candesartan, and fluoxetine [5].

The selection of drugs was based on the database of BfArM (Bundesinstitut für Arzneimittel und Medizinprodukte), where pharmaceutical companies may report supply shortages of their products [6]. Additionally, the “Gelbe Liste” [7] was used to collect information about supply shortages. Drugs were classified according to the drug groups used by the “Rote Liste” [8]. Further information regarding the BfArM database, the Gelbe Liste and Rote Liste can be found in the supplementary file.

For the survey, drugs were selected from groups with the highest frequency of reports in the BfArM register and the Gelbe Liste. Consequently, the groups of antibiotics, psychotropic agents, analgesics, antihypertensives, antiasthmatic drugs, cytotoxic drugs, and antidiabetics were chosen. Antiepileptic drugs were excluded from the survey, as no reports for this group were registered in the Gelbe Liste.

The survey questions referred to the time period from November 2022 to January 2024 and consisted of four blocks of questions:

1. General inquiries: Federal state of the workplace, area of specialization, and work setting of the survey participant.
2. Impact of supply shortages: How participants were affected by the shortages of the 20 drugs.
3. Chosen alternative measures: Further questions regarding the drugs that had the greatest impact on participants’ work including which alternative measure was chosen most often.
4. Time consumption: Measuring the increased time consumption resulting from supply shortages.

The survey is anonymous and data protection compliant, with only 3 personal questions that do not allow for allocation of responses to individual participants. The overview of the questionnaire can be found in the supplementary file Table S2. The complete questionnaire can be found as supplementary table.

A scale of 1–6 was chosen to evaluate the success of treatment, with 1 corresponding to very poor treatment success of the alternative compared to the originally intended medication and 6 corresponding to very good treatment success.

### General information about the realization of the survey

The online questionnaire was created using SoSci Survey and made available to participants at www.soscisurvey.de. The licence holder is Dr Dominik Leiner from SoSci Survey GmbH in Munich, Germany [9]. The survey was conducted using the program, which was licensed by the “Medizinische Hochschule Hannover”. The survey was launched on January 7, 2024, and closed on August 4, 2024. Intended participants included physicians practicing in Germany and Austria. The survey is aimed at all medical disciplines, which is why general medical associations and associations of statutory health insurance physicians were contacted. In addition, contact was made with associations of general practitioners and associations of physicians specialising in internal medicine, as these disciplines (apart from physicians without a specialisation) have the largest membership [10]. A total of 75 associations of physicians in Germany and 26 in Austria were contacted. Ultimately, 18 German and 9 Austrian associations responded to the survey and assisted in its dissemination (see supplementary file Fig. S5, Fig. S6).

### General information about the analysis of the survey

The analysis of the survey response data has been performed in Python within a Jupyter Notebook utilizing pandas, numpy and matplotlib. Incomplete answers to question blocks were excluded. No further inferential statistics have been used.

**Fig. 1:**
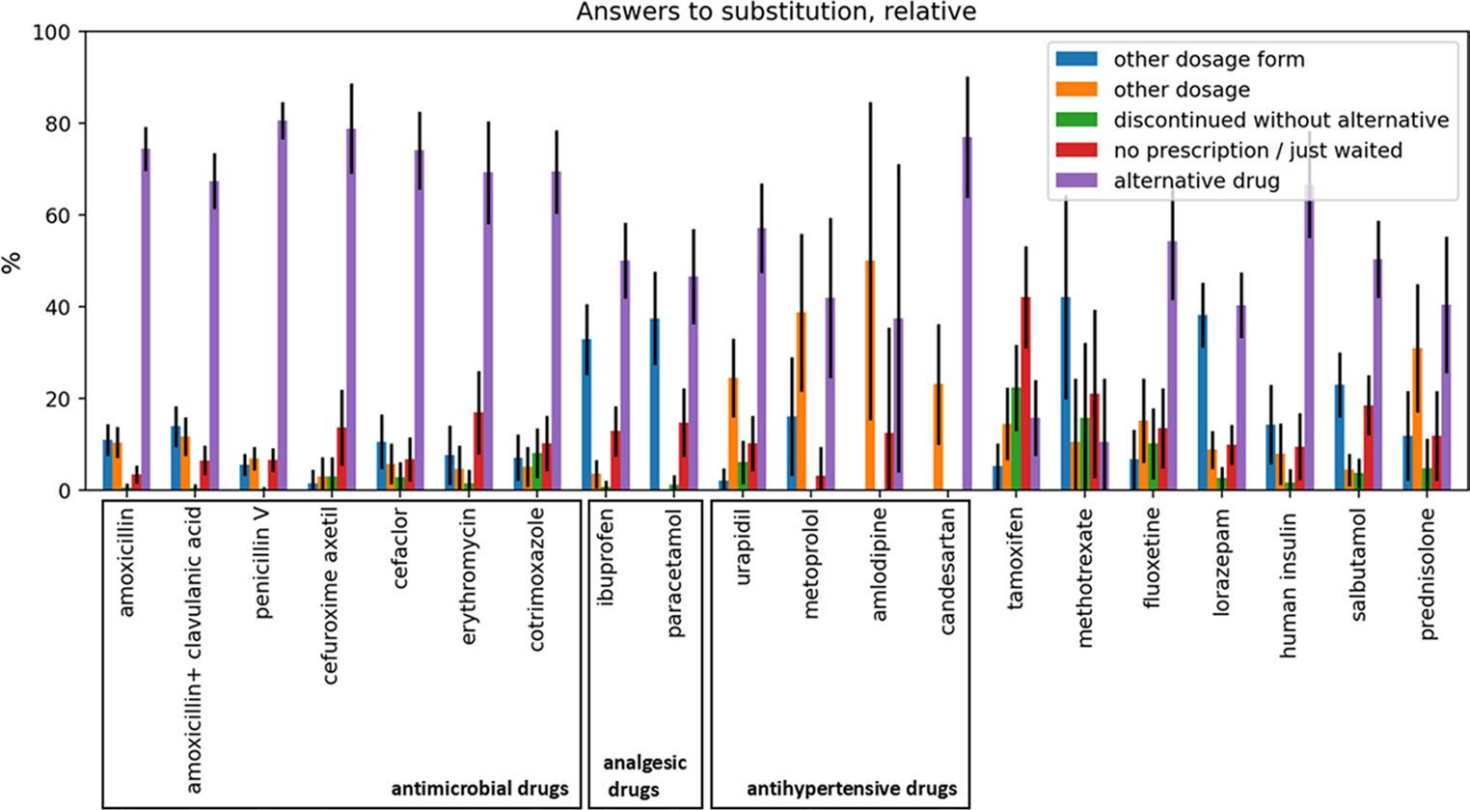
Distribution of answers to the 5 different ways of substitution for each of the 20 drugs. Survey question: “How did you deal with the supply shortage of the drug *XYZ* with the dosage form selected (in the question before) for the majority of your patients?” The confidence interval of 95% is described as a vertical black line in each column

The confidence interval of 95% in Figs. 1 and 3 is calculated based on the margin of error:

$$\:{MOE}_{95}\approx\:1.96\:\sqrt{\frac{{\sigma\:}^{2}}{n}}$$

## Results

In total 895 physicians participated and were analysed.

665 survey participants stated to be practicing in Germany and 190 survey participants stated to be practicing in Austria. The survey participants from Austria were predominantly based in Upper Austria, with half of the participants practicing in this region. Physicians from the other federal states also participated, but to a much smaller extent. In contrast, the survey participants from Germany were most frequently based in Lower Saxony (54%), followed by Thuringia (25%). Notably, every federal state was represented among the German participants, although some states only with small numbers.

More than half of the participants were general practitioners. Internal specialists and paediatricians formed the second and third largest groups.

The shortage of the selected drugs lasted 11 months or more during the observed period, except for ibuprofen. Detailed data on the duration of the shortage and the number of reported medicines is provided in the supplementary file Fig. S1, S2, S3 and S4. Additionally; in Table S1 in the supplementary file further information on the selection of drugs for the survey is provided.

### How physicians coped with the supply shortages of the drugs

For the antibacterials included in the survey, physicians most frequently (at least 67%) chose an alternative drug in the event of a shortage of the respective drug (Fig. 1). The proportion of physicians who chose one of the other alternative measures was significantly lower. For the analgesic drugs, ibuprofen and paracetamol, however, the choice of other alternative measures was more frequently reported. 33% (ibuprofen) and 38% (paracetamol) of physicians stated that they had chosen a different dosage form as an alternative. In addition, 13% (ibuprofen) and 15% (paracetamol) of survey participants stated that they had initially waited without a replacement instead of using the unavailable drug. Compared to antibacterials and analgesics, a larger proportion of physicians chose a different dosage of the same drug when replacing antihypertensives. In the case of tamoxifen, the largest proportion of survey participants (42%) stated that they had initially waited without prescribing a drug.

The total number of responses to the question refers to the number of survey participants who answered the question about their choice of alternative drug for the respective drug (original drug).

Colour coding of the drugs in Table 1: Classification of antibacterials based on their spectrum of activity and their risk of antimicrobial resistance using the WHO Access, Watch, Reserve (AWaRe) classification 2025 [11].

**Table 1:**
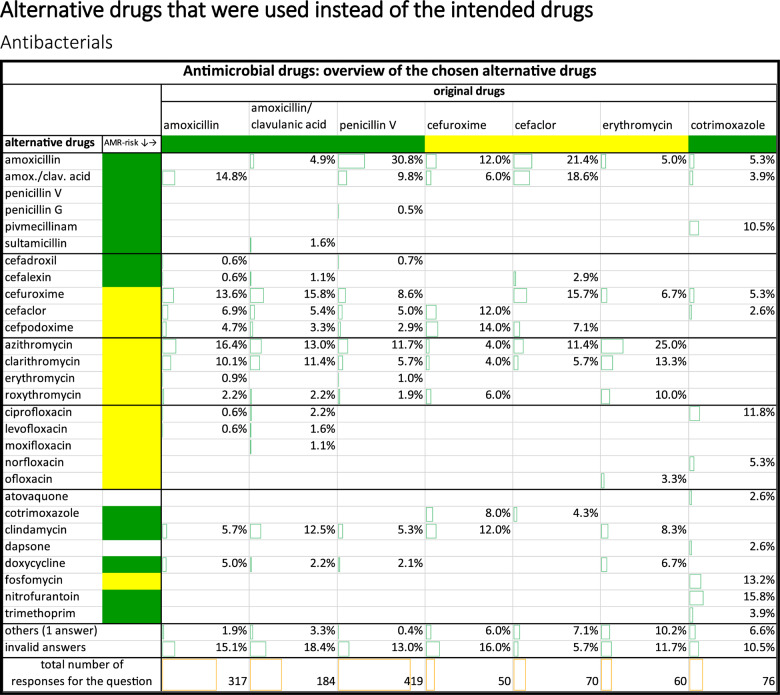
Alternative antibacterial drugs selected. Original drugs: The drug that the physicians would have chosen without a supply shortage for the substance. Alternative drugs: The drug that the physicians chose for most of their patients instead of the unavailable original drug


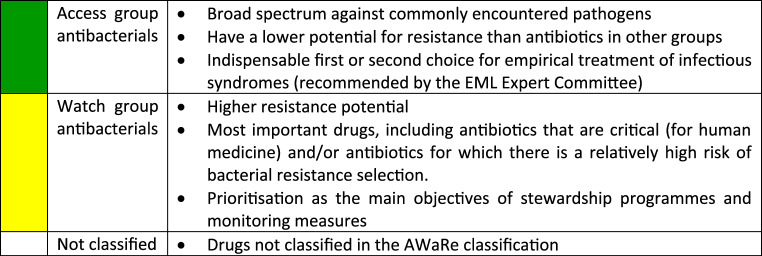


Drugs in the Access group are often narrow-spectrum antibacterials, whereas those in the Watch group have a broad spectrum of activity [12].

Amoxicillin was most commonly replaced with azithromycin and amoxicillin-clavulanic acid. However, unlike amoxicillin and amoxicillin-clavulanic acid, azithromycin is classified in the ‘Watch’ group rather than the ‘Access’ group. In doing so, drugs from the ‘watch’ group, which have a higher potential for resistance, were used instead of the originally intended drugs from the ‘access’ group. Amoxicillin/clavulanic acid was most commonly replaced with cefuroxime or azithromycin. Both alternatives are part of the Watch group, unlike amoxicillin/clavulanic acid. Penicillin V was largely replaced by amoxicillin. However, penicillin V was also replaced by a cephalosporin or a macrolide. The most commonly chosen cephalosporins and macrolides are part of the watch group. The cephalosporins cefuroxime (watch group) and cefaclor (watch group) were largely replaced by a penicillin (access group) or another cephalosporin (watch group). Erythromycin (access group) has largely been replaced by other macrolides such as azithromycin (watch group). Cotrimoxazole has largely been replaced by nitrofurantoin (access group) and fosfomycin (watch group). In general, when replacing antibacterials that were unavailable due to supply shortages, an alternative from the ‘Watch’ group – with a broad spectrum of activity and a higher potential for resistance – was frequently chosen instead of an antibacterial from the ‘Access’ group.”

####  Other drugs form the survey

**Fig. 2:**
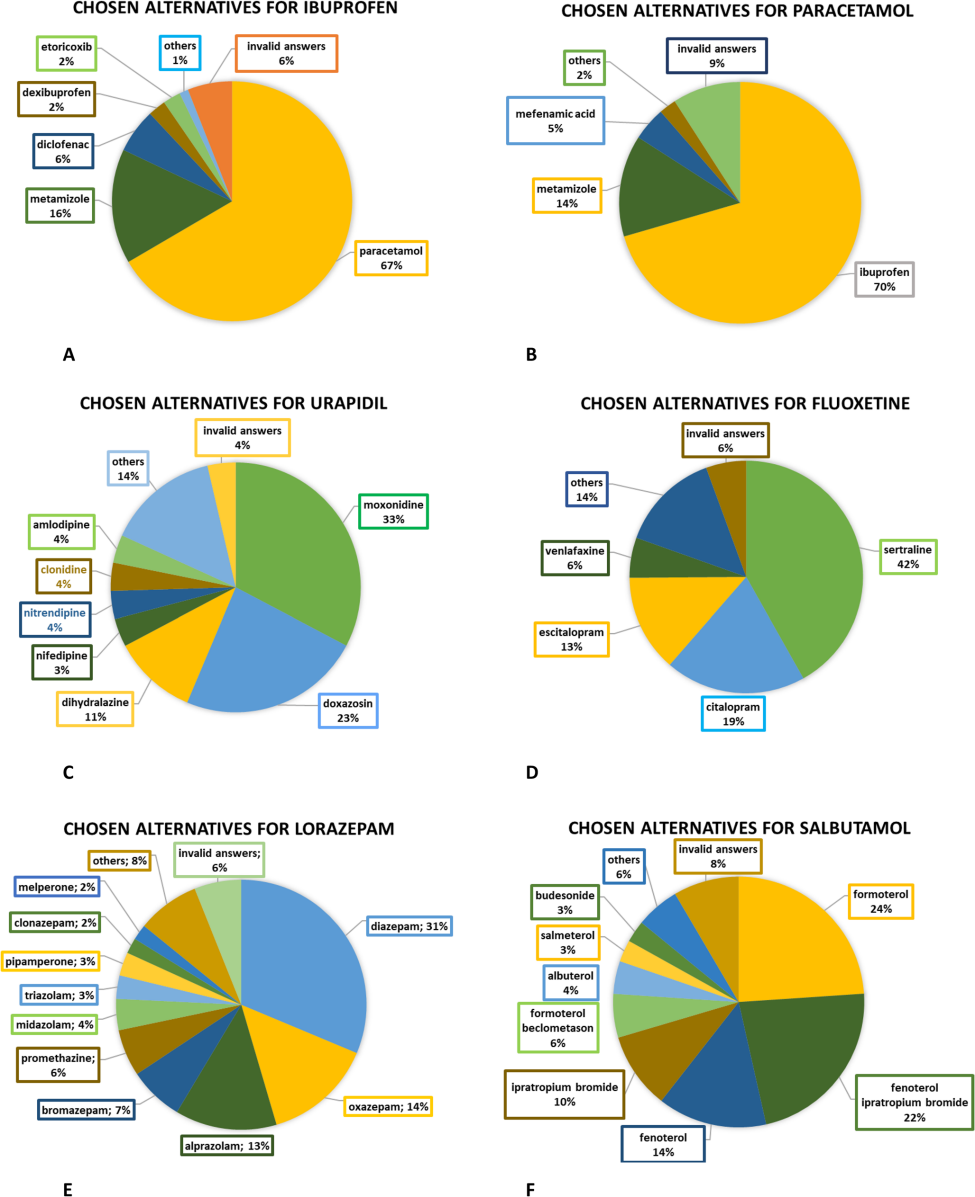
(**A**,** B**,** C**,** D**,** E**,** F**): Chosen alternative drugs for the drugs from the survey. Survey question: Which alternative did you choose instead of ibuprofen (**A**)/paracetamol (**B**)/ urapidil (**C**)/fluoxetine (**D**)/lorazepam (**E**)/ salbutamol (**F**) most of the time? The label “others” summarizes all alternative drugs that were mentioned only once in the survey results. Total number of answers: **A**: 84; **B**: 44; **C**: 55, **D**: 36; **E**: 99, **F**: 71

67% of physicians replaced ibuprofen with paracetamol (Fig. 2 A). Ibuprofen was also used as an alternative to paracetamol by 70% of survey participants (Fig. 2B). Both ibuprofen and paracetamol were most commonly replaced with metamizole (Fig. 2 A, B). Urapidil was mostly replaced with moxonidine (by 33% of survey participants) and doxazosin (by 23% of survey participants) (Fig. 2 C). 42% of physicians chose sertraline as an alternative to fluoxetine. Citalopram was chosen by 19% of physicians as an alternative to fluoxetine (Fig. 2D). Lorazepam was mainly replaced with diazepam (by 31% of physicians), oxazepam (by 14% of physicians) and alprazolam (by 13% of physicians). 24% of survey participants chose formoterol instead of salbutamol (Fig. 2E). Fenoterol with and without ipratropium bromide was the second most frequently chosen alternative to salbutamol (Fig. 2 F).

No statement can be made about the alternative drugs of the other drugs that were queried in the survey because of a too low response rate.

### Rating the treatment success

Overall, there were no significant differences between the responses of physicians practicing in Germany and physicians practicing in Austria when comparing the survey data.

**Fig. 3:**
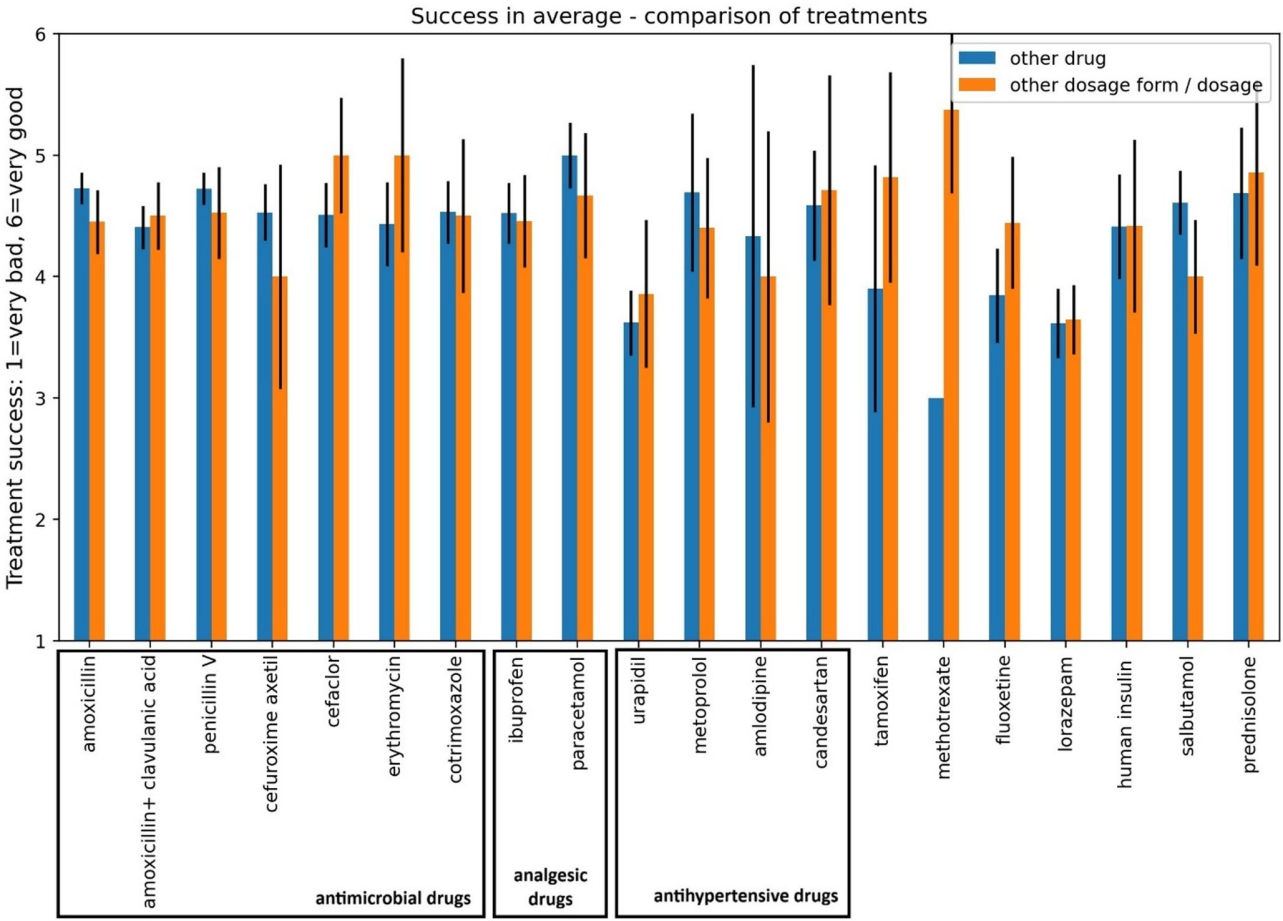
Success in average comparing the treatment “other drug” and “other dosage form/dosage for each of the 20 drugs. Treatment success from 1 to 6: 1 = very poor in comparison to the originally intended drug, 6 = very good in comparison to the originally intended drug. The confidence interval of 95% is described as a vertical line in each column

In order to analyse the extent of which the outcome for the patient has changed, the treatment success of the alternatives, from the point of view of the physicians, has been evaluated in the survey (Fig. 3).

The treatment success of the selected alternatives was evaluated on a scale of 1–6. 1 meant that the treatment success of the alternative was very poor compared to the originally selected drug, while 6 meant that the treatment success was very good. Firstly, it can be seen that for most drugs there is no significant discrepancy between the two alternatives ‘other drug’ and ‘other dosage form/dosage’. The treatment success for most drugs was rated above 4. The exceptions here are urapidil and lorazepam, for which the treatment success of the alternatives was rated below 4.

## Discussion

### Consequences of drug shortages for patients based on the survey results

#### Change in dosage

In the case of antihypertensive drugs, more than 20% of survey participants reported switching to a different dosage of the same drug in most instances. This may involve altering the number of tablets taken by patients. When the dosage of antihypertensive drugs is changed, close monitoring of blood pressure may also be necessary. In a cross-sectional secondary data analysis using data from telephone interviews, it was analysed how patients implemented complex medication regimens. It showed that 48.1% of patients made one or more dosing errors [13]. Changes in dosages could therefore lead to additional confusion and thus incorrect drug dosages by patients.

#### Change in dosage form

In the case of ibuprofen and paracetamol, the dosage form was often changed. As the syrup was most often reported to be unavailable for both drugs, this can force children to have to swallow tablets instead of having a syrup-based dosage form. Solid oral dosage forms, even with antibacterial drugs, are not well tolerated by toddlers and preschoolers [14].

#### No prescription/just waited

The shortages of tamoxifen were often addressed by pausing tamoxifen therapy without an alternative or by delaying the start of therapy with no prescription and a wait-and-see approach. Tamoxifen, a selective oestrogen receptor modulator, is used to treat postmenopausal and premenopausal breast cancer in women [15]. However, discontinuing tamoxifen therapy is associated with a higher mortality rate in women with early breast cancer [16]. The BfArM recommends replacing tamoxifen, but these alternatives are often associated with more adverse effects. Aromatase inhibitors in combination with GnRH analogues are an example of an alternative to tamoxifen, whereas suspension of therapy without an alternative is not recommended [17]. A retrospective cohort study and survey of physicians on oncological drugs revealed that alternative drugs had a greater toxicity in 34.8% of cases [18], highlighting the consequences of omitting or substituting oncological drugs.

#### Modification of the drug prescription

##### Antibacterials

When selecting alternative antibacterials, particular attention should be paid to their potential for resistance. Some of the selected alternatives exhibit a higher potential for resistance than the originally intended drug. This is evident from the fact that antibacterials classified in the Access Group in the AWaRe classification have been replaced with antibacterials from the Watch Group. Consequently, the antibacterials shown may have contributed to a higher risk of antimicrobial resistance developing due to supply shortages. The consequences of uncontrolled antimicrobial resistance (AMR) are severe, with 39.1 million deaths predicted to be attributable to AMR, and 169 million deaths associated with AMR from 2025 to 2050 [19]. By 2030, at least 65% (compared to 62.8% in 2024) of total antibiotic consumption should consist of Access-antibacterials. This is the aim of the German Antibiotic Resistance Strategy (DART-2030) [20, 21]. However, the drug shortages outlined here could hinder the achievement of this target.

##### Analgesics

The second most commonly chosen alternative to paracetamol and ibuprofen was metamizole (Fig. 2AB). The comparison of alternative drugs for ibuprofen and paracetamol highlights the fragility of the system, as the best alternatives for these drugs are also subject to supply shortages. This creates a vicious cycle, where the absence of one drug leads to the absence of its alternatives, exacerbating the shortage. The reliance on metamizole as a second-line alternative further complicates the situation, as it may not be sufficient to compensate for the shortages of two drugs (ibuprofen and paracetamol) (Table 2AB).

**Table 2:** List of the originally intended drugs, their most commonly chosen alternatives, and the problems arising from their substitution

Original intended drug	most frequently used alternative	Problems that may arise as a result of the replacement	Explanation of the replacement problems
amoxicillin	azithromycin (Table 1)	Different resistance potential Different adverse drug reactions	• Amoxicillin is classified in the Access Group (according to the AWaRe classification) and has been replaced by azithromycin from the Watch Group, which increases the risk of antimicrobial resistance developing [16] • When azithromycin is taken alongside drugs that prolong the QT interval, the risk of cardiac events is 40% higher compared with amoxicillin [22].
amoxicillin/ clavulanic acid	cefuroxime (Table 1)	Different resistance potential Different adverse drug reactions	• Amoxicillin/clavulanic acid is classified in the Access Group and has been replaced by cefuroxime from the Watch Group, which increases the risk of antimicrobial resistance developing [16] • In a population-based, nested case-control study from 2002 and 2022 from Ontario Canada it was found out, that cephalosporins and sulphonamide antibiotics carried the highest absolute risks of serious cADRs (cutaneous adverse drug reactions) [23].
penicillin V	amoxicillin (Table 1)	Larger spectrum of activity More impact on the gut microbiome	**Penicillin V** • Very narrow antimicrobial spectrum. Effective against Gram-positive bacteria such as S. pneumoniae and Group A streptococci. • Due to its limited effect on Gram-negative bacteria, it has only a minimal impact on the gut microbiome [24]. **Amoxicillin**: • Narrow spectrum of activity. Effective against Gram-positive bacteria (S. pneumoniae and Group A streptococci). Also effective against selected Gram-negative bacteria, particularly non-β-lactamase-producing H. influenzae. • Also effective against many commensal bacteria (residing in the gut microbiome), leading to overgrowth of Enterobacteriaceae species and the selection of antibiotic-resistant strains [24]
cefuroxime	cefpodoxime (Table 1)	Risk of insufficient therapy because of different spectra of activity	**Cefuroxime (second-generation cephalosporin)**: • effective against Gram-positive cocci and certain Gram-negative rods [25] **Cefpodoxime (third-generation cephalosporin)**: • Effective against Haemophilus influenzae and most Enterobacterales, e.g. *Escherichia coli*,* Klebsiella pneumoniae* and *Proteus mirabilis*, which do not produce AmpC beta-lactamases or extended-spectrum beta-lactamases (ESBLs). Also effective against some Gram-positive species, particularly streptococci, including some strains with reduced penicillin sensitivity [25].
cefaclor	amoxicillin (Table 1)	Different spectra of activity	**Cefaclor (second-generation cephalosporin)**: • effective against Gram-positive cocci and certain Gram-negative rods [25] **Amoxicillin**: • effective against enterococci and certain Gram-negative bacteria such as *Haemophilus influenzae*,* E. coli*,* Proteus mirabilis*,* Salmonella spp.* and Shigella spp. that do not produce beta-lactamase [25].
erythromycin	azithromycin (Table 1)	Differences for pregnant patients	Erythromycin is considered safer during pregnancy than azithromycin, due to its more frequent use and the resulting greater understanding of its effects [25].
cotrimoxazole	nitrofurantoin (Table 1)	Different spectra of activity	**Cotrimoxazole**: • effective against a broad spectrum of Gram-positive bacteria (including some strains of methicillin-*resistant Staphylococcus aureus*). Also effective against a broad spectrum of Gram-negative bacteria, the protozoa *Cystoisospora* and *Cyclospora spp.*, and the fungus *Pneumocystis jirovecii* [25]. **Nitrofurantoin**: • effective against uropathogenic pathogens such as *Escherichia coli*,* Staphylococcus saprophyticus* and *Enterococcus faecalis* [25]
Ibuprofen	paracetamol (Fig. 2 A)	Different mechanisms of action, contraindications and adverse drug reactions	**Ibuprofen** • Non-selective cyclooxygenase inhibitor • Clinical use: Wide-ranging; inhibition of inflammatory processes, pain relief, reduction of fever • Contraindications: Renal impairment, gastrointestinal ulcers, known hypersensitivity reactions, pregnancy, increased cardiovascular risk, impaired blood clotting [26] • adverse drug reactions: o Erosions and ulcers of the gastric and intestinal mucosa o Effect on renal blood flow: in patients with pre-existing kidney damage, there is a risk of acute kidney failure o Hypersensitivity reactions o Increased cardiovascular mortality [26] **Paracetamol**: • Mechanism of action not fully understood • Analgesic, antipyretic; **no anti-inflammatory effect** • Contraindications: Severe liver and kidney damage and glucose-6-phosphate dehydrogenase deficiency. Caution is advised in cases of chronic alcoholism (liver!) [26] • adverse drug reactions: o at therapeutic doses, non-specific adverse drug reactions (e.g. hypersensitivity reactions) are very rare [26]
paracetamol	Ibuprofen (Fig. 2B)		
urapidil	moxonidine (Fig. 2 C)	Different indications	**Urapidil**: • Alpha-1 adrenergic receptor antagonist • Indications: essential hypertension, benign • prostatic hyperplasia, as an adjunct vasodilator in heart failure, and possibly in Raynaud’s syndrome **Moxonidin**: • Alpha-2 adrenergic receptor agonist • Indications: hypertension [26] The shared indication for both drugs is hypertension. According to the hypertension guidelines, neither of these drugs is a first-line treatment for hypertension. Urapidil is recommended as the first-line treatment for pre-operative pheochromocytoma. Urapidil is also administered intravenously in hypertensive emergencies. These indications do not apply to moxonidine [27]. It is therefore clear that the indications for the two drugs differ.
fluoxetine	sertralin (Fig. 2D)	Different indications	Fluoxetine is used to treat depression [28], obsessive-compulsive disorder [29], and bulimia nervosa [30]. Notably, fluoxetine has unique characteristics that make it difficult to replace. It is the only SSRI approved for treating depression in children [31] and is also the only SSRI approved in Germany for treating bulimia nervosa [32]. Additionally, fluoxetine has a longer half-life (4–6 days) compared to sertraline (1 day) and citalopram (1.5 days), which makes it particularly effective for a longer period [33]. For this reason, a switch to fluoxetine is normally also recommended for dosing in the event of discontinuation and withdrawal phenomena [34]. But in case of supply shortage of fluoxetine, it cannot be used in such occasions.
lorazepam	diazepam (Fig. 2E)	Differences in the duration of action	Notably, lorazepam was not always replaced with a benzodiazepine with a similar half-life. Diazepam has a longer half-life (20–100 h) compared to lorazepam (5–20 h) [29]. Therefore, diazepam may exhibit a stronger overhang effect the next day compared to lorazepam when used to treat insomnia.
salbutamol	formoterol (Fig. 2 F)	Differences in the duration of action	Salbutamol and formoterol are both ß2 adrenoreceptor agonists [29]. Salbutamol belongs to the subgroup of SABA (short acting beta agonist) and formoterol to the subgroup of LABA (long-acting beta agonist) [29]. The two drugs are therefore effective for different lengths of time. It is therefore interesting here that salbutamol was not necessarily replaced with a drug from the same group (SABA).

##### PRISCUS List

The PRISCUS List contains 177 drugs which should not be used, or should only be used with particular caution, in the pharmacotherapy of older and multimorbid patients [35]. This list of potentially inappropriate medications (PIMs) was compiled in collaboration with the German Medical Association’s Drug Commission and other project partners. The survey responses show that drugs not included on the PRISCUS list have been replaced with drugs from the PRISCUS list (e.g. paracetamol replaced with ibuprofen, or urapidil with moxonidine). This poses a greater risk to older patients [36].

#### Treatment success

For most drugs, the treatment success is rated similarly between “different dosage form or dosage” and “different drug” within the margin of error. However, the treatment success of alternative treatments for urapidil and lorazepam was rated worse, both for “different dosage form or dosage” and “different drug” (Fig. 3). The tablet form of urapidil was the most frequently missing dosage form, and as a reserve antihypertensive drug [29], it is not regularly used unless blood pressure cannot be adjusted with other antihypertensive drugs. This may lead to poorer treatment success due to the manipulation of an already unstable system.

The poorer treatment success of alternative therapies for lorazepam may be attributed to the dosage form. The most frequently missing dosage forms were tablets or orodispersible tablets, and the selected alternative drugs such as diazepam, oxazepam and alprazolam are only approved in oral tablet form in Germany [37]. Orodispersible tablets have the advantage of a faster onset of action compared to oral tablets [38]. If the improvement of anxiety or restlessness due to the lack of alternative orodispersible forms is delayed for the patient, this may have a negative effect on the success of the treatment. For the other drugs, the treatment success was rated < 5 out of 6, indicating a poorer outcome than expected with the original [39, 40].

### Further effects of supply shortages on patients and society

#### Medication errors

Medication errors are more common when prescribing practices are shifted to lesser-known alternative drugs, especially if they are less effective, have a poorer profile of adverse drug reactions, or require an unusual or difficult dosing regimen [41]. Such alternatives are resorted to due to supply shortages and therefore medication errors can occur more frequently. Furthermore, medication errors were reported to lead to death in one in 131 outpatients and in one in 854 inpatients [42]. In the survey of 193 pharmacy managers, respondents also reported one to five undesirable events that were likely or possibly related to drug shortages. The majority of respondents reported 1–10 medication errors due to omissions and dispensing/administering the wrong dose/drug [43].

#### Prolonged hospital stays

A survey by the European Association of Hospital Pharmacists (EAHP) in 2018 found that 20% of 946 participants reported a longer hospital stay for affected patients due to supply shortages [44]. A similar survey in 2023 found that almost 30% of 627 hospital pharmacists confirmed this impact on patients [39]. Both surveys also indicated a delayed start of therapy as a result of supply shortages [39, 44], which can also lead to an extended hospital stay. Additionally, inferior alternative treatments due to drug shortages [18] could provoke prolonged hospital stays. Even in the survey some of the alternative measures were rated with < 4 out of 6 which could result in an inferior alternative treatment due to drug shortage.

#### Patient mortality

In a scoping review in 2019, the medical outcome in the event of supply shortage was examined. In 10 out of 16 studies, increased mortality due to supply shortages was found. Drug groups associated with a poor outcome for the patient included antibacterials and oncological drugs [40].

#### Financial consequences

According to a Report to Congress by the Advisory Body of the Secretary of the U.S. Department of Health and Human Services (ASPE= Office of the Assistant Secretary for Planning an Evaluation) in Washington (DC) the price of drugs with a supply shortage increased by 16.6%. Alternative drugs showed a price increase three times higher than those of drugs with a supply shortage [45]. Especially in the case of supply shortage of generics, an increase in the price of the drugs is associated with the shortage [46]. During a supply shortage in China, the price of drugs increased by 27.57% between 2019 and 2021, compared to an annual price increase of 11.62% for drugs without supply shortages [47]. A survey of hospital pharmacists in Europe found that 24% of respondents confirmed increased costs for patients due to drug shortages, while 60% confirmed the use of more expensive alternatives. Additionally, 50% of survey participants reported rising pharmacy costs or personnel costs due to supply shortages [48].

### Limitations

Firstly, a response bias emerges due to the predominance of participants from the field of general medicine. The distribution of the specialisations in this survey is therefore not the same as the distribution in reality. Due to the uneven distribution, the results may be distorted. Secondly, it can be inferred that physicians who were severely impacted by supply shortages were more likely to participate in this survey, whereas those who were minimally affected or unaffected by the mentioned shortages may have been underrepresented. Thirdly, the survey’s reliance on participant recollections of past events introduces the potential for recall bias and, if applicable, reporting bias. Fourthly, the fact that not all participants completed the questionnaire contributes to a loss to follow-up bias. Fifthly, the uneven distribution of participants across states may also influence the survey’s results.

## Conclusions

The survey provides an overview of alternative therapy measures and their consequences as rated by the physicians due to the surveyed supply shortages of 20 drugs. This assessment is based on the subjective evaluation of practicing physicians in Germany and Austria. For many of the selected affected drugs, it was possible to choose the original drug in a different dosage form or dosage. However, for antibacterials, most physicians had to choose alternative drugs, which may increase the risk of therapeutic failure or resistance development. Notably, the treatment success of urapidil and lorazepam was rated worse than that of other drugs. For the other drugs, the treatment success was rated < 5 out of 6, indicating a poorer outcome than expected with the original drug. Future projects should investigate treatment success in more detail, including measuring the use of selected alternatives for a certain clinical entity over a longer period of time and comparing it with the use of the unavailable drug. The focus of this survey was on the subjective assessment of physicians to extract serious differences in treatment success. Since April 2020, a task force has been established in Germany to ensure drug supply in intensive care units, involving various stakeholders to create a reliable list of crucial drugs and develop a model procedure to ensure supply hotspots. Additionally, concrete demand and production capacities were determined for Germany, and measures were developed to avoid supply problems in intensive care treatment [49]. Furthermore, medical staff should be trained to handle future supply shortages, and physicians should be assisted in choosing alternative drugs to minimize adverse drug reactions and medication errors.

## Supplementary Information

Below is the link to the electronic supplementary material.

Supplementary file (DOCX 467 KB)

Supplementary table (PDF 430 KB)

## Data Availability

All source data of this study are available from the authors upon reasonable request.

## Abbreviations

RKI: Robert Koch Institute
BfArM: Bundesinstitut für Arzneimittel und Medizinprodukte (Federal Institute for Drugs and Medical Devices)
MHH: Medizinische Hochschule Hannover (Hanover medical school)
AMR: antimicrobial resistance
