# Supplementary material for: Drug supply shortages and their perceived consequences for patients: a questionnaire survey of German and Austrian physicians
Source: Eur J Clin Pharmacol. 2026 May 1;82(5):138. doi: 10.1007/s00228-026-04052-4 (PMC13132925; doi:10.1007/s00228-026-04052-4)
Supplement: Supplementary file 1 — Supplementary file (DOCX 467 KB) [file 228_2026_4052_MOESM1_ESM.docx]

**Supplementary file for
“Drug supply shortages and their far-perceived consequences for patients: A questionnaire survey for German and Austrian physicians”**

**Julia Maria Rotter and Roland Seifert***

**Institute of Pharmacology, Hannover Medical School,**

**Carl-Neuberg-Straße 1, D-30625, Hannover, Germany**

***Corresponding author:** [**seifert.roland@mh-hannover.de**](mailto:seifert.roland@mh-hannover.de)

For Figure 1-4 it should be noted that not all supply shortages must necessarily be reported in the BfArM (Bundesinstitut für Arzneimittel und Medizinprodukte) database where pharmaceutical companies may report supply shortages of their products [1]. Pharmaceutical companies self-committed to report the following supply shortages of medicines: [2]

1. Medicinal products containing a medicinal substance relevant for patient supply and provided by a single source only [2]
2. Medicinal products with prescription drugs that have a market share of 25% or more
3. Medicinal products that are subject to the obligation to report to hospitals in accordance with section 52b (3a) of the AMG (Arzneimittelgesetz/German Medicines Act) [2]

**
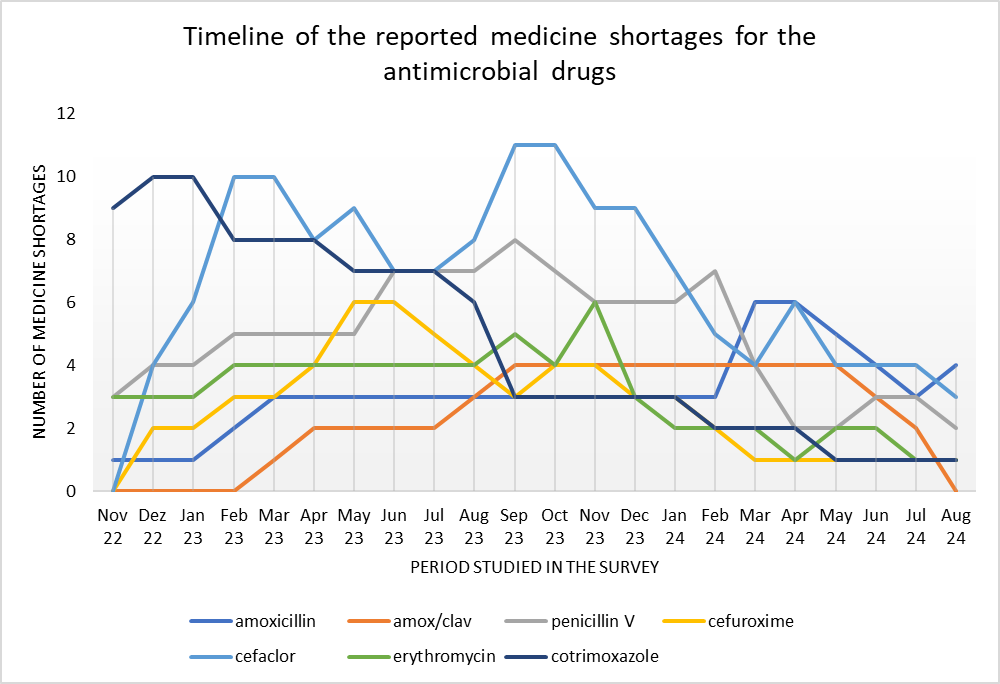
**

**Fig. S1:** Timeline of reported shortages for antibacterial medicinal substances

The diagram shows drug shortages reported to the BfArM (status as of October 14, 2025) [1] for the 7 antibacterial medicinal substances questioned in the survey; amox/clav: amoxicillin/clavulanic acid;

**
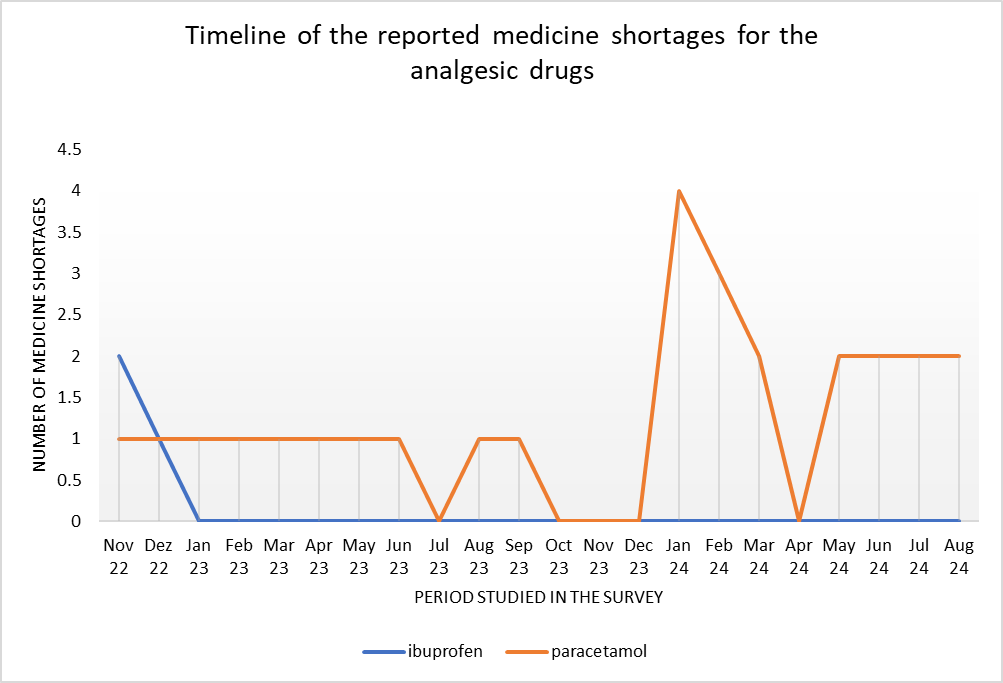
**

**Fig. S2:** Timeline of reported shortages for analgesic medicinal substances

The diagram shows drug shortages reported to the BfArM (status as of October 14, 2025) [1] for the two analgesic medicinal substances questioned in the survey;

**
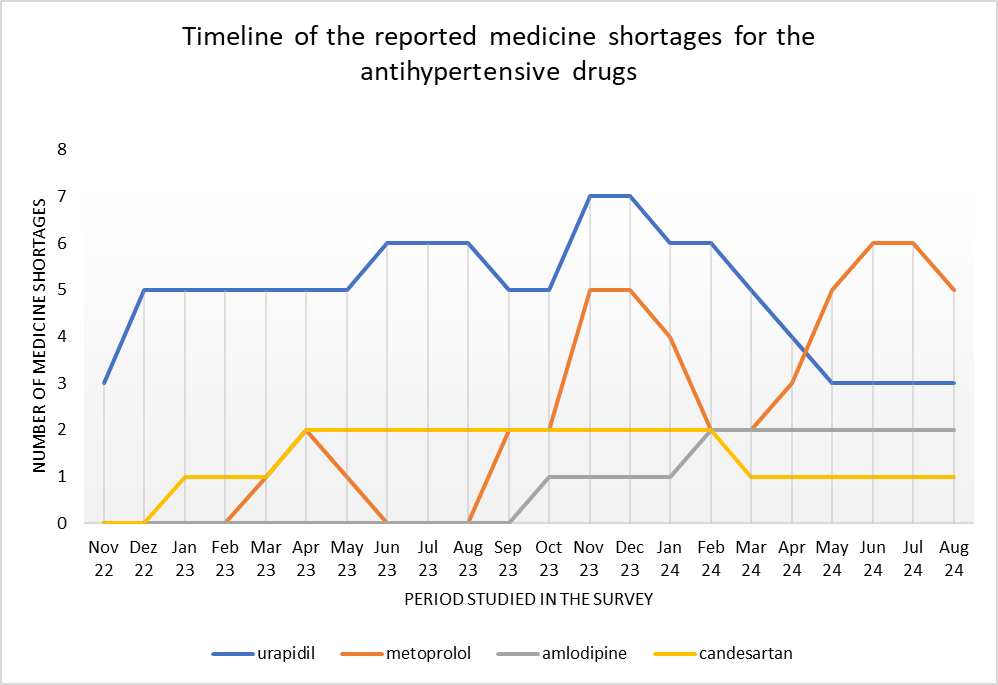
**

**Fig. S3:** Timeline of reported shortages for antihypertensive medicinal substances

The diagram shows drug shortages reported to the BfArM (status as of October 14, 2025) [1] for the 4 antihypertensive medicinal substances questioned in the survey

**
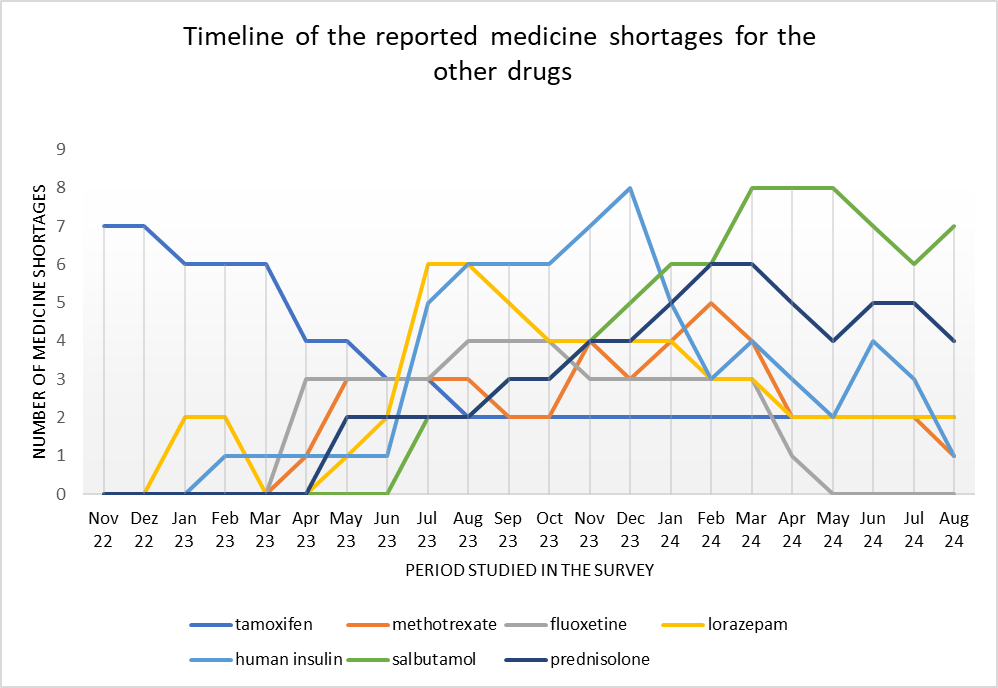
**

**Fig. S4:** Timeline of reported shortages for the other medicinal substances

The diagram shows drug shortages reported to the BfArM (status as of October 14, 2025) [1] for the 7 other medicinal substances questioned in the survey

| **Drug groups** | **Chosen drug** | **Justifikation** |
| --- | --- | --- |
| antibacterial drugs | amoxicillin | Most drug shortage reports for antibacterials in BfArM and the Gelbe Liste |
|  | Amoxicillin + clavulanic acid |  |
|  | penicillin V |  |
|  | cefuroxime | Cephalosporins with the most drug shortage reports in the BfArM |
|  | cefaclor |  |
|  | erythromycin | Macrolide with the most drug shortage reports in the BfArM together with azithromycin. More reports of erythromycin were relevant to supply. |
|  | cotrimoxazole | Representative for inhibitors of tetrahydrofolic acid synthesis (antibiotic group with the most reports apart from the groups already mentioned) |
| analgesics/ antirheumatics | ibuprofen | 1 drug shortage report in BfArM and in Gelbe Liste  -> selected because of extensive media presence [3] |
|  | paracetamol | 3 drug shortage reports in BfArM and 0 in Gelbe Liste ->selected because of extensive media presence [4] |
| antihypertensive drugs | urapidil | Most drug shortage reports for antihypertensives in BfArM |
|  | metoprolol | Representing group b [5] |
|  | amlodipine | Representing group c [5] |
|  | candesartan | Representing group a [5] |
| cytotoxic drugs, other antineoplastic agents and protective agents | tamoxifen | Most notifications in BfArM for cytostatic drugs |
|  | methotrexate | In comparison to the other drug with the most notifications named topotecan, it is not only used for the treatment of cancers. In addition, low-dose methotrexate is used in the treatment of rheumatoid arthritis. [6] |
| psychotropic drugs | fluoxetine | According to the Professional Association for Child and Adolescent Psychiatry, Psychosomatics and Psychotherapy in Germany ([BKJPP),](https://www.kinderpsychiater.org/startseite/) no other medicinal substance is approved for children and adolescents for the treatment of severe forms of depressive disorders. [7] |
|  | lorazepam | Reported in BfArM and Gelbe Liste |
| antidiabetic drugs | human insulin | Most reports for antidiabetic drugs |
| bronchospasmolytic/ antiasthmatics and other respiratory tract medications/ corticoids | salbutamol | The most frequently mentioned drugs from the Rote Liste Group 28; 31 |
|  | prednisolone |  |

**Table S1**: Selection criteria of the 20 drugs from the 7 groups [8] with the most notifications [9] [10]

First, a list of all reported supply shortages was exported from the BfArM database [1]This list was then narrowed down to supply shortages in the period under review (November 2022 to the beginning of 2024). From the groups with the most supply shortage reports, the drugs with the most supply shortage reports were then selected. All shortages reported during the period under review were also filtered out on the Gelbe Liste website, and the drugs with the most supply shortages were also filtered out. The results from the two sources were then compared and the following list of drugs was compiled. Reports from the BfArM were given priority, as this is the official reporting office for supply shortages in Germany and can therefore be assumed to be the most complete.

Explanation of the registers used:
Gelbe Liste:

The Gelbe Liste Online provides medical news, information and databases for doctors, pharmacists and other healthcare professionals. The free online service of Vidal MMI Germany GmbH has an editorial team consisting exclusively of experts such as doctors, pharmacists and medical journalists. Editorial content and advertising are clearly separated from each other. The Gelbe Liste Pharmindex Drug Database is based on officially approved product and use information, which is published by the respective manufacturers and prepared by the Gelbe Liste Pharmindex editorial team according to the four-eyes principle [11].

Rote Liste:

For over 90 years, the Rote Liste has been a reliable, impartial and advertising-free source of information on medicines in Germany [12].

| **Question number** | **Question** | **Answers** |
| --- | --- | --- |
| **Question block 1 General questions** | | |
| 1.1 | Enter the state of your place of work | Drop-Down menu  with federal states of Germany and Austria |
| 1.2 | Select your field of study | Drop-Down menu  with specializations |
| 1.3 | In which setting do you work? | practice  clinic  company  other workplace |
| **Question block 2**  **Individual evaluation of all 20 drugs** | | |
| 2.1 | Please assess the extent to which you have been affected by supply shortage for the following drugs | Rating each of the 20 drugs with a number from 1 to 5  1=not affected  2=slightly affected  3=moderately affected  4=strongly affected  5=very strongly affected  I can't judge |
| **Question block 3**  **Enquiries about evaluation in 2.1**  All drugs present in 2) **rated >= 3** are supplemented with the following questions per drug  "For the drug *XYZ,* you have selected 3 or higher; please answer the following questions only in relation to the drug ***XYZ***" | | |
| 3.1 | 3.1) Which dosage form of drug XYZ was most affected? | Choosing a dosage form |
| 3.2 | How did you deal with the supply shortage of the drug ***XYZ*** with the dosage form selected in 3.1 for the largest number of your patients? | - I have prescribed a different dosage form of the drug **XYZ** ." - I have prescribed a different dosage of the drug XYZ - I discontinued the drug XYZ earlier as planned without an alternative - I did not prescribe the drug from the beginning and waited - I have prescribed a different drug |
| 3.3 | Which alternative drug(s) have you chosen? | Free text field |
| 3.4 | Evaluate the treatment success of the alternative compared to the originally intended drug. | 1-6 points*  1=very poor  6=very good |
| **Question block 4**  **Time spent due to supply shortages** | | |
| 4.1 | Please indicate how much additional time you had for the respective tasks per drug concerned  Tasks:  Telephone calls with the pharmacy  Search for alternatives for the Pat.  Conversation/Enlightenment of the Pat. | Evaluation of the 3 tasks with one of the time indications:  Approx. 0min per drug  Approx. 5min per drug  Approx. 10min per drug  Approx. 15min per drug  Approx. >=20min per drug |
| 4.2 | Did you have any additional time expenditure due to something else that has not yet been mentioned above? | Free text field to give another reason for additional time expenditure |

**Table S2**: Questionnaire of the survey divided into 4 question blocks

*Since there is no 0 as a response code in soSci survey (the program that was used for the survey), the scale of 1-6 was used in the evaluation from the queried scale of 0-5.

This paper focuses on question block 3, as considering all the results would go beyond the scope of this paper. The answers to the questions in block 1 can be found in figures 5, 6 and 7 below. In summary, the questions in block 2 revealed concern about the shortages of penicillin V and amoxicillin. The most additional time expenditure (question block 4) was reported by physicians in haematology/oncology, psychiatry, nephrology and general medicine. A detailed examination of the effects of drug shortages on the medical profession will be addressed in a separate paper.


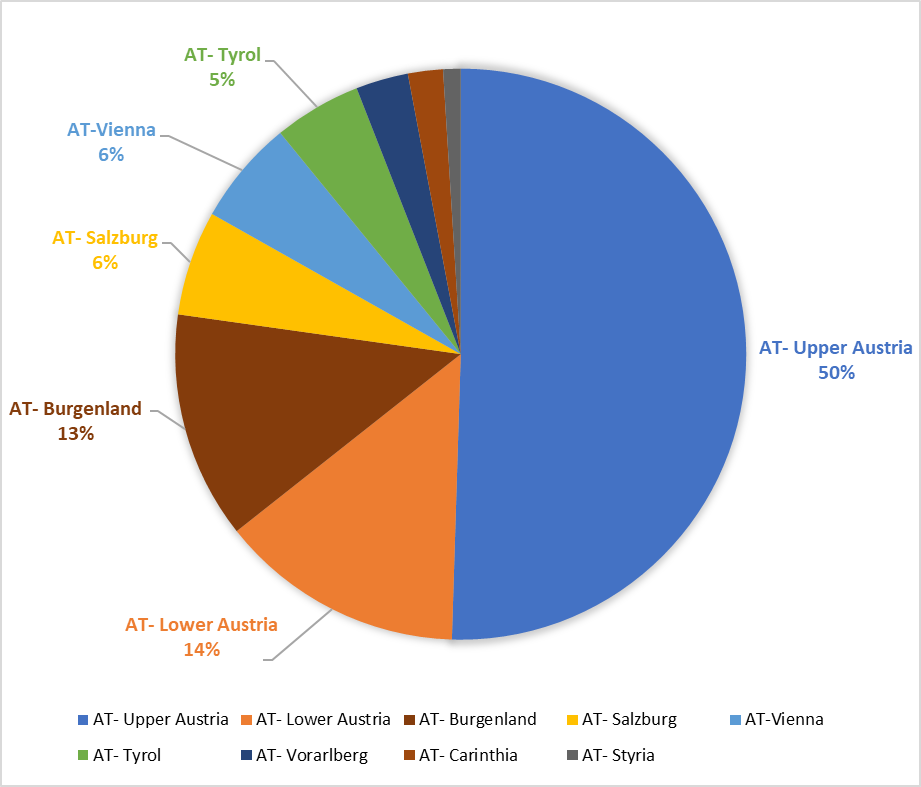


**Figure S5:** Distribution of survey participants from Austria by federal state

**
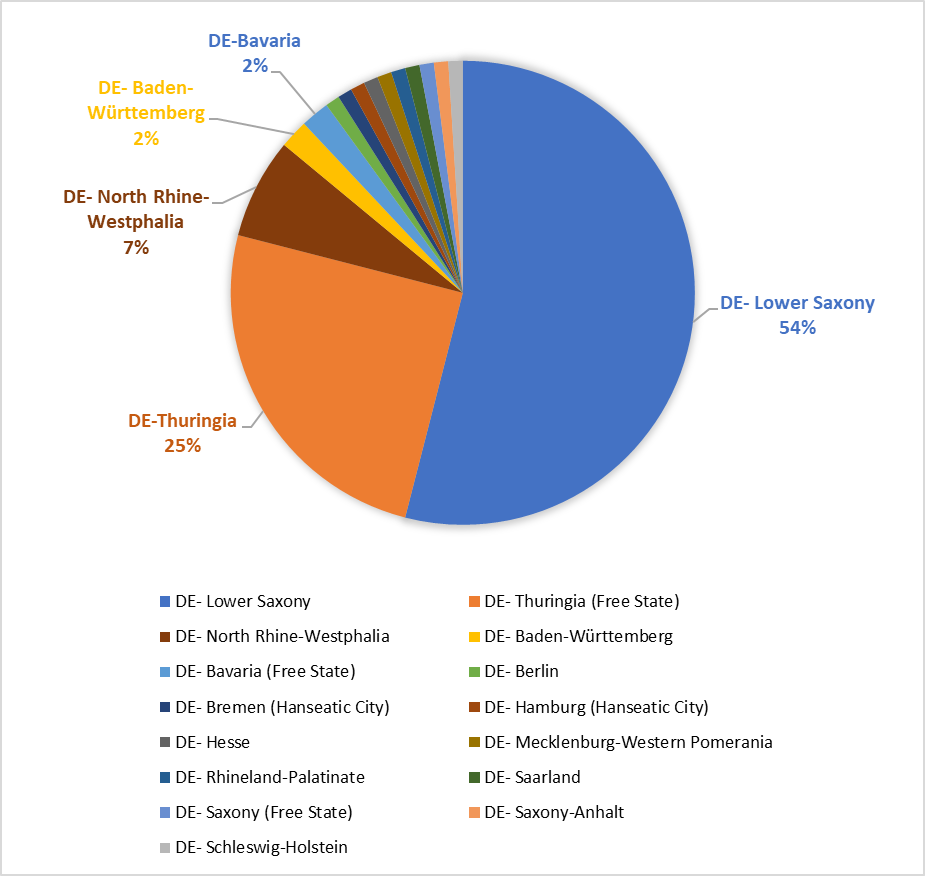
**

**Figure S6:** Distribution of survey participants from Germany by federal state.


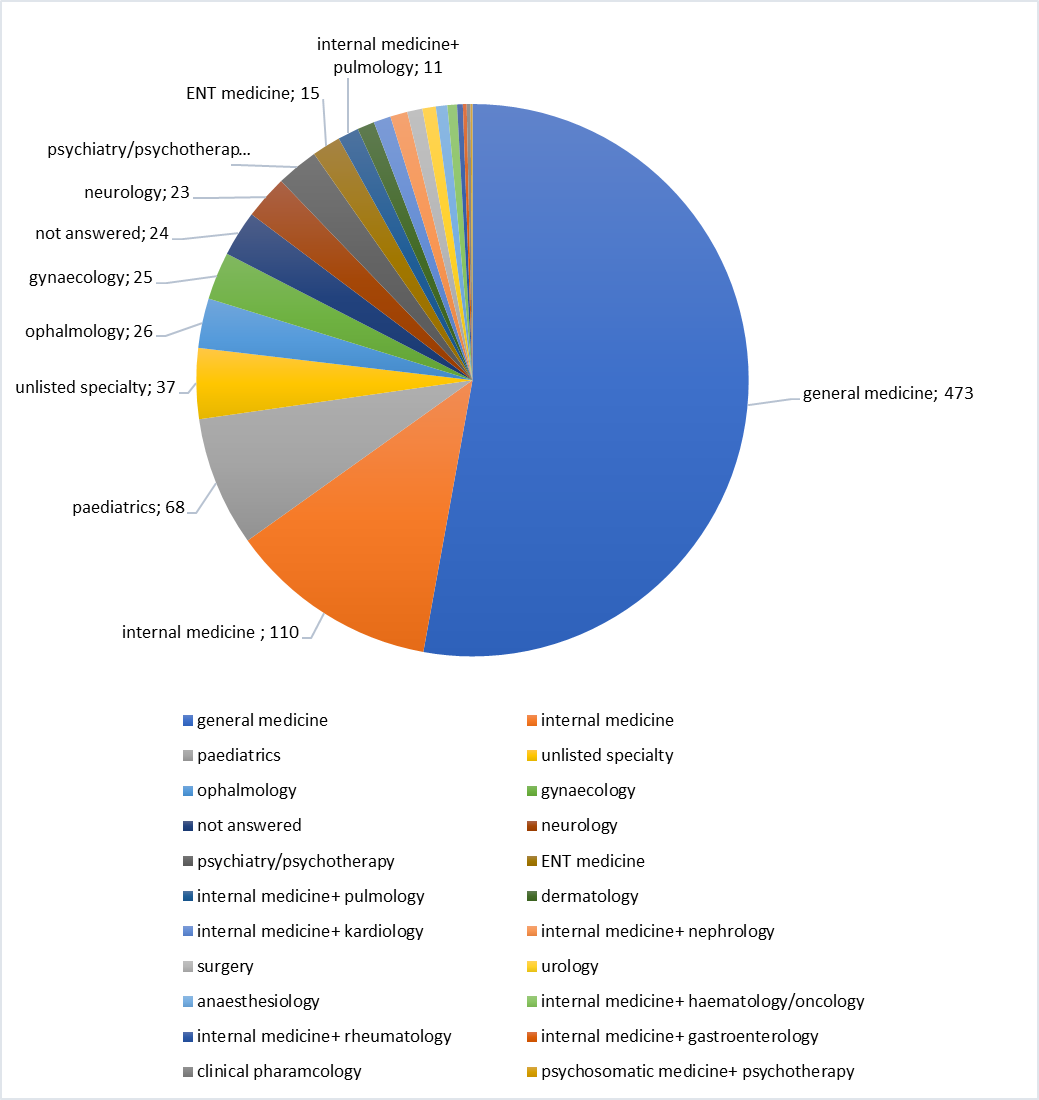


**Figure S7:** The number of survey participants for each specialty

| **German survey distributors** | **Austrian survey distributors** |
| --- | --- |
| Ärztekammer (German medical chamber)   - Thüringen (Thuringia) - Saarland - Westfalen-Lippe - Bremen (Hanseatic City) - Schleswig-Holstein - Mecklenburg-Vorpommern (Mecklenburg-Western Pomerania) | Österreichische Ärztekammer  (Austrian medical chamber)   - Burgenland - Oberösterreich (Upper Austria) - Salzburg |
| Kassenärztliche Vereinigung (association of Statutory Health Insurance Physicians)   - Hamburg (Hanseatic City) - Schleswig- Holstein - Mecklenburg-Vorpommern  (Mecklenburg-Western Pomerania) - Niedersachsen (Lower Saxony) - Sachsen (Saxony (Free State)) | Vereinigung österreichischer Ärzte  (Association of Austrian medical doctors) |
| Hausärzteverband Westfalen-Lippe  Hausärzteverband Saarland  Hausärzteverband Niedersachsen | - Österreichisches Institut für Allgemeinmedizin Kärnten (öifam) (Austrian institute for general medicine Carinthia) - Tiroler Gesellschaft für Allgemeinmedizin (TGAM) (Tyrolean Society for General Medicine) - Oberösterreichische Gesellschaft für Allgemeinmedizin (Obgam) (Upper Austrian Society for General Medicine) |
|  | Der Arzneimittelbrief (the medication letter) |
| Deutsche Gesellschaft für Innere Medizin  (German society for internal medicine) |  |
| Berufsverband Deutscher Internistinnen und Internisten  (Professional association of German internists) |  |
| Institut für Allgemeinmedizin und Palliativmedizin der Medizinischen Hochschule Hannover  (Institute for general and palliative medicine at the MHH |  |

**Table S3**: List of German and Austrian survey distributors

**Reference List**

1. Bundesinstitut für Arzneimittel und Medizinprodukte (BfArM) (2025) Veröffentlichte Lieferengpassmeldungen. [https://anwendungen.pharmnet-bund.de/lieferengpassmeldungen/faces/public/meldungen.xhtml. Accessed 14.10 2025](https://anwendungen.pharmnet-bund.de/lieferengpassmeldungen/faces/public/meldungen.xhtml.%20Accessed%2014.10%202025)

2. Bundesinstitut für Arzneimittel und Medizinprodukte (2025) Meldeverpflichtungen für Pharmazeutische Unternehmen. https://www.bfarm.de/DE/Arzneimittel/Arzneimittelinformationen/Lieferengpaesse/Meldeverpflichtungen/_node.html. Accessed 17. Nov. 2025

3. Deutsche Apotheker Zeitung (2023) 90.000 Packungen Ibuprofen-Suspension aus der Ukraine. <https://www.deutsche-apotheker-zeitung.de/news/artikel/2023/01/04/90-000-packungen-ibuprofen-suspension-aus-der-ukraine>. Accessed: 29. Aug.2025

4. Deutsches Ärzteblatt (2022) Lieferengpass: Paracetamolhaltige Fiebersäfte betroffen. <https://www.aerzteblatt.de/archiv/lieferengpass-paracetamolhaltige-fiebersaefte-betroffen-b16fd88b-b269-4002-8e78-c03292e25acc>. Accessed: 29. Aug.2025

5. Seifert R (2018) Basiswissen Pharmakologie, 1. Auflage. Springer- Verlag GmbH Deutschland, Hannover

6. Freissmuth M, Offermanns S, Böhm S (2024) Pharmakologie und Toxikologie Von den molekularen Grundlagen zur Pharmakotherapie, 4. Auflage. Springer Verlag GmbH, Wien, Frankfurt, Wien

7. Deutsches Ärzteblatt (2023) Lieferengpass bei einzigem Antidepressivum für Kinder und Jugendliche. https://www.aerzteblatt.de/news/lieferengpass-bei-einzigem-antidepressivum-fuer-kinder-und-jugendliche-20468c65-f643-47bc-8d6b-9eb0450f8cd0. Accessed: 21 Apr. 2025

8. Rote Liste (2025) Rote Liste Arzneimittelinformationen für Deutschland. https://www.rote-liste.de/. Accessed: 21 Mar. 2025.

9. Bundesinstitut für Arzneimittel und Medizinprodukte (BfArM) (2024) Veröffentlichte Lieferengpassmeldungen. <https://anwendungen.pharmnet-bund.de/lieferengpassmeldungen/faces/public/meldungen.xhtml>. Accessed 12.01 2025

10. Gelbe Liste (2023) Lieferengpässe bei Medikamenten. https://www.gelbe-liste.de/lieferengpaesse/lieferengpaesse-medikamente. Accessed: 5 Nov. 2023

11. Vidal MMI Germany GmbH (2026) GELBE LISTE. PHARMINDEX. <https://www.gelbe-liste.de/>. Accessed 28. Jan. 2026

12. Rote Liste (2026) Über Rote Liste. <https://rote-liste.com/%C3%BCber-uns>. Accessed 18 Mar. 2026
