# Supplementary material for: Drug supply shortages and their perceived consequences for patients: a questionnaire survey of German and Austrian physicians
Source: Eur J Clin Pharmacol. 2026 May 1;82(5):138. doi: 10.1007/s00228-026-04052-4 (PMC13132925; doi:10.1007/s00228-026-04052-4)
Supplement: Supplementary file 2 — Supplementary table (PDF 430 KB) [file 228_2026_4052_MOESM2_ESM.pdf]

Supplementary table

for “Drug supply shortages and their far-perceived consequences for patients:
A questionnaire survey for German and Austrian physicians”

Julia Maria Rotter and Roland Seifert*

Institute of Pharmacology, Hannover Medical School,
Carl-Neuberg-Straße 1, D-30625, Hannover, Germany

*Corresponding author: seifert.roland@mh-hannover.de

The survey was written and conducted in German. However, the survey text has also been translated into English below (blue text).

Seite 01
page 01

Einführungstext

Sehr geehrte Ärztinnen und Ärzte,
vielen Dank, dass Sie sich bereiterklären an der Umfrage zum Thema Lieferengpässe von Medikamenten im Zeitraum von November 2022 bis Anfang 2024 teilzunehmen.

Sämtliche von Ihnen in der Online-Befragung gemachten Angaben werden vertraulich und unter Einhaltung der gesetzlichen Datenschutzbestimmungen gemäß der europäischen Datenschutzgrundverordnung (DSGVO) behandelt. Die Antworten im Fragebogen werden ausschließlich im Rahmen des Projekts der Promotionsarbeit zu Lieferengpässen verwendet. Personenbezogene Daten werden nicht an Dritte und an der Untersuchung nicht beteiligte Personen bzw. Institutionen weitergegeben. Die Teilnahme an der Befragung ist freiwillig und anonym. Rückschlüsse auf die Personen der Teilnehmer sind nicht möglich und auch nicht beabsichtigt. Sollten Sie dennoch keine persönlichen Angaben zur Ihrem Arbeitsort etc. machen wollen, gibt es bei den entsprechenden Fragen die Möglichkeit, "Keine Angabe" auszuwählen.

Bitte beantworten Sie die Fragen ehrlich. Es gibt keine falschen Antworten. Aus Gründen der leichteren Lesbarkeit wird auf eine geschlechtsspezifische Differenzierung, wie z.B. Patient/innen, verzichtet. Entsprechende Begriffe gelten im Sinne der Gleichbehandlung für alle Geschlechter.

Wenn Sie die Umfrage an Ihrem Handy machen, halten Sie es am besten im Querformat.

Bei Fragen oder zum Widerruf Ihrer Daten können Sie sich gerne an
julia.m.rotter@stud.mh-hannover.de
wenden.

Introductory text

Dear physicians,
Thank you for agreeing to participate in the survey on drug supply shortages from November 2022 to early 2024. All information you provide in the online survey will be treated confidentially and in compliance with the legal data protection provisions of the European General Data Protection Regulation (GDPR). The answers in the questionnaire will be used exclusively within the scope of the doctoral thesis project on supply shortages. Personal data will not be passed on to third parties or persons not involved in the study or institutions not involved in the study. Participation in the survey is voluntary and anonymous. It is not possible or intended to draw conclusions about the participants as individuals. However, if you do not wish to provide any personal information about your place of work, etc., you have the option of selecting ‘not specified’ for the relevant questions. Please answer the questions honestly. There are no wrong answers. For reasons of ease of readability, gender-specific distinctions have been omitted.
Corresponding terms apply to all genders in the spirit of equal treatment.
If you are completing the survey on your mobile phone, it is best to hold it in landscape format.
If you have any questions or wish to revoke your data, please feel free to contact
julia.m.rotter@stud.mh-hannover.de.

Geben Sie das Bundesland Ihres Arbeitsortes an: [Bitte auswählen] [Please select]

Please specify the state in which you work

1. Wählen Sie Ihre Fachrichtung aus:

[Bitte auswählen] [Please select]

Select your medical specialisation

2. In welchem Setting arbeiten Sie?

What is the setting in which you work?

- ○ Praxis — doctor's office
- ○ Klinik — clinic
- ○ Firma — company
- ○ anderer Arbeitsplatz — other workplace
- ○ Keine Angabe — not specified

3. In welchem Ausmaß waren Sie von Lieferengpässen der folgenden Wirkstoffe betroffen? To what extent were you affected by supply shortages for the following drugs?

Zu den 5 höchstbewerteten Wirkstoffen werden weitere Fragen gestellt.
Further questions are asked about the five highest-rated drugs.

		nicht betroffen not affected	leicht betroffen mildly affected	mittelmäßig betroffen moderately affected	stark betroffen severely affected	sehr stark betroffen very severely affected	kann ich nicht beurteilen I cannot judge
Amoxicillin	amoxicillin	◯	◯	◯	◯	◯	◯
Amoxicillin/ Clavulansäure	amoxicillin/ clavulanic acid	◯	◯	◯	◯	◯	◯
PenicillinV (Phenoxymethylpenicillin)	penicillin V/ phenoxymethyl-penicillin	◯	◯	◯	◯	◯	◯
Cefuroximaxetil	cefuroxime	◯	◯	◯	◯	◯	◯
Cefaclor	cefaclor	◯	◯	◯	◯	◯	◯
Erythromycin	erythromycin	◯	◯	◯	◯	◯	◯
Cotrimoxazol	cotrimoxazole	◯	◯	◯	◯	◯	◯
Ibuprofen	ibuprofen	◯	◯	◯	◯	◯	◯
Paracetamol	paracetamol	◯	◯	◯	◯	◯	◯
Urapidil	urapidil	◯	◯	◯	◯	◯	◯
Metoprolol	metoprolol	◯	◯	◯	◯	◯	◯
Amlodipin	amlodipine	◯	◯	◯	◯	◯	◯
Candesartan	candesartan	◯	◯	◯	◯	◯	◯
Tamoxifen	tamoxifen	◯	◯	◯	◯	◯	◯
Methotrexat	methotrexate	◯	◯	◯	◯	◯	◯
Fluoxetin	fluoxetine	◯	◯	◯	◯	◯	◯
Lorazepam	lorazepam	◯	◯	◯	◯	◯	◯
Insulin human	human insulin	◯	◯	◯	◯	◯	◯
Salbutamol	salbutamol	◯	◯	◯	◯	◯	◯

Prednisolon prednisolone	○	○	○	○	○	○

Seite 04
page 04

Im Folgenden werden Ihnen Nachfragen gestellt zu den Lieferengpässen der Wirkstoffe, die Sie besonders hoch bewertet haben.

Below, you will be asked questions about the supply shortages for the drugs that you rated particularly highly.

Seite 05
page 05
w01

Amoxicillin

Die nächsten 4 Fragen beziehen sich nur auf den Wirkstoff Amoxicillin.
Bitte beantworten Sie folgende Fragen nur im Hinblick auf den Lieferengpass dieses Wirkstoffs.
The next 4 questions relate only to the drug amoxicillin.
Please answer the following questions only with regard to the supply shortage of this drug.

4. Welche Darreichungsform des Amoxicillin war aus Ihrer Sicht am meisten betroffen? In your opinion, which dosage form of amoxicillin was most affected?

- ◯ Tabletten (Sublingualtablette, Schmelztablette) tablets (sublingual tablets, orodispersible tablets)
- ◯ Dragee / Kapsel dragee/ capsule
- ◯ Salbe / Creme / Gel ointment/cream/gel
- ◯ Tropfen drops
- ◯ Sirup / Saft syrup/juice
- ◯ Injektionslösung injection solution
- ◯ Infusionslösung infusion solution
- ◯ Brausetablette effervescent tablet
- ◯ Pflaster plaster
- ◯ Spray spray
- ◯ Zäpfchen suppository

5. How did you deal with the supply shortage of amoxicillin, in the dosage form you had just chosen, for most of your patients?

5. Wie sind Sie mit dem Lieferengpass von Amoxicillin mit der gerade eben gewählten Darreichungsform bei den meisten Ihrer Patienten umgegangen?

- ◯ „Ich habe eine andere Darreichungsform von Amoxicillin verschrieben" I have prescribed a different dosage form of amoxicillin.
- ◯ „Ich habe eine andere Dosierung von Amoxicillin verschrieben" I have prescribed a different dosage of amoxicillin.
- ◯ „Ich habe Amoxicillin ohne alternativen Wirkstoff vorzeitig abgesetzt" I stopped taking amoxicillin prematurely without an alternative drug.
- ◯ „Ich habe Amoxicillin gar nicht erst verschrieben und erst einmal ohne alternativen Wirkstoff abgewartet" I did not prescribe amoxicillin in the first place and waited without an alternative drug.
- ◯ „Ich habe einen alternativen Wirkstoff verschrieben" I have prescribed an alternative drug.

6. Welche(n) alternative(n) Wirkstoff(e) haben Sie anstelle von Amoxicillin gewählt? Which alternative drug(s) did you choose instead of amoxicillin?

(Falls Sie in der vorherigen Frage „Ich habe einen alternativen Wirkstoff verschrieben" ausgewählt haben, sonst können Sie die Frage einfach frei lassen) If you selected 'I have prescribed an alternative drug' in the previous question, otherwise you can simply leave the question blank.

7. Bewerten Sie den Behandlungserfolg der Alternative im Vergleich zum ursprünglich gewollten Medikament Evaluate the treatment success of the alternative compared to the originally intended medication

0=sehr schlecht very poor

5= sehr gut very good

○ 0 ○ 1 ○ 2 ○ 3 ○ 4 ○ 5

Für die 5 Arzneistoffe mit der in Frage 3 angegebenen höchsten Betroffenheit, wurde den an der Umfrage Teilnehmenden die Fragen 4, 5 und 6 angezeigt. Hier sind diese Fragen exemplarisch nur für Amoxicillin dargestellt, um die Übersichtlichkeit des Fragebogens zu wahren. Die Seiten, auf denen die gleichen 3 Fragen für die anderen Arzneistoffe stehen, wurden daher an dieser Stelle exkludiert.

For the five drugs for which the survey participant stated to be affected the most in question 3, questions 4, 5 and 6 were displayed to survey participants. For the sake of clarity, these questions are shown here only for amoxicillin. The pages containing the same three questions for the other drugs have therefore been excluded at this point.

84. Geben Sie bitte an, wie viel Zeitmehraufwand Sie für die jeweiligen Aufgaben hatten pro betroffenen Wirkstoff

Please indicate how much additional time you spent on each task per drug affected.

Telefonate mit der Apotheke

0 Min pro Wirkstoff	ca. 5 Min. pro Wirkstoff	ca.10min pro Wirkstoff	ca.15min pro Wirkstoff	ca. >=20min pro Wirkstoff
○	○	○	○	○

time: 0->=20 minutes per drug

Suche nach Alternativen

0 Min pro Wirkstoff	ca. 5 Min. pro Wirkstoff	ca.10min pro Wirkstoff	ca.15min pro Wirkstoff	ca. >=20min pro Wirkstoff
○	○	○	○	○

time: 0->=20 minutes per drug

Gespräch/Aufklärung des Patienten

0 Min pro Wirkstoff	ca. 5 Min. pro Wirkstoff	ca.10min pro Wirkstoff	ca.15min pro Wirkstoff	ca. >=20min pro Wirkstoff
○	○	○	○	○

time: 0->=20 minutes per drug

85. Hatten Sie noch einen Zeitmehraufwand durch etwas anderes, das oben noch nicht genannt wurde?

Did you have to spend extra time on anything else that has not been mentioned above?

Wenn ja, schreiben Sie bitte den Grund für den Zeitmehraufwand in die Zeile

If so, please write the reason for the additional time required in the line

Vielen Dank für Ihre Teilnahme! Thank you for participating.

Wir möchten uns ganz herzlich für Ihre Mithilfe bedanken. We would like to thank you very much for your help.

Ihre Antworten wurden gespeichert, Sie können das Browser-Fenster nun schließen.
Your answers have been saved. You may now close the browser window.

Julia Rotter – 2024
